# Resistance Mechanisms in Immunotherapy-Radiotherapy/Chemotherapy Combinations in Locally Advanced Head & Neck Squamous Cell Carcinoma

**DOI:** 10.32604/or.2026.077918

**Published:** 2026-09-14

**Authors:** Abbas Hussain, Daniel Thomas Jones, Rishi Kumar Nanda, Ramaditya Srinivasmurthy, Jason Ta, Yin Mon Myat, Riccesha Hattin, Jo-Lawrence Bigcas, Sisi Tian, Suparna Shah, Robert Wang, Kyaw Zin Thein

**Affiliations:** 1 Department of Internal Medicine, Kirk Kerkorian School of Medicine at UNLV, Las Vegas, NV, USA; 2 Department of Internal Medicine, Sunrise Health GME Consortium, Las Vegas, NV, USA; 3 College of Osteopathic Medicine, Touro University Nevada, Las Vegas, NV, USA; 4 Department of Internal Medicine, Mount Sinai Morningside/West, Mount Sinai, New York, NY, USA; 5 Department of Internal Medicine, HCA Healthcare/USF Morsani College of Medicine GME: HCA Florida Citrus Hospital, Inverness, FL, USA; 6 Department of Internal Medicine, One Brooklyn Health: Interfaith Medical Center Campus, Brooklyn, NY, USA; 7 Department of Otolaryngology-Head & Neck Surgery, University of Nevada, Las Vegas, NV, USA; 8 Division of Hematology and Medical Oncology, Comprehensive Cancer Centers of Nevada, Las Vegas, NV, USA

**Keywords:** Locally advanced head and neck squamous cell carcinoma (LA-HNSCC), combination immunotherapy strategies, radiosensitization and immunotherapy, immunotherapy resistance, precision immuno-oncology, translational oncology, tumor microenvironment (TME)

## Abstract

Locally advanced head and neck squamous cell carcinoma (LA-HNSCC) remains difficult to treat despite multimodal therapy. Immune checkpoint inhibitors (ICIs) have expanded treatment options, but phase III trials combining ICIs with chemoradiotherapy have demonstrated limited survival benefit due to complex resistance mechanisms. These include immunosuppressive tumor microenvironments, impaired DNA damage responses, hypoxia-driven adaptations, metabolic reprogramming, and oncogenic signaling via the HER receptor family. This review outlines key resistance pathways and emerging strategies to overcome them. Nanotechnology-based approaches may enhance drug delivery and modulate the tumor microenvironment, while dual inhibition of epidermal growth factor receptor (EGFR), human epidermal growth factor receptor 3 (HER3), and downstream pathways shows promise in addressing compensatory signaling. Advances in biomarkers, including programmed death-ligand 1 (PD-L1), circulating tumor DNA, metabolic profiling, and radiomics, enable improved patient selection and monitoring. Novel immune checkpoints and adoptive cellular therapies, alongside personalized and adaptive trial designs, offer potential to improve outcomes in LA-HNSCC.

## 1 Introduction

Head and neck cancers (HNC) represent a substantial global health challenge, accounting for about 700,000 new cases and over 350,000 deaths reported annually [1]. About 30–40% of early-stage disease (stage I or II) HNC patients are curable and experience improved survival rates after surgery or radiotherapy alone. In contrast, more than 60% of advanced-stage (stage III or IV) HNC patients need advanced therapeutic options [2]. Despite the utilization of aggressive multimodal treatment modalities, using combinations of surgery and chemoradiation therapies, the overall 5-year survival for patients with HNC remains modest at 40–50%, while for recurrent/metastatic disease, the median survival is only around 10 months [3,4]. The integration of immunotherapy, notably immune checkpoint inhibitors (ICIs has greatly impacted the management of head and neck cancers, with some randomized controlled trials demonstrating significant clinical benefit in a subset of patients [5]. However, treatment resistance—particularly in combined immunotherapy and radiotherapy/chemotherapy regimens—remains a major barrier to improving outcomes in locally advanced HNC.

The efforts to incorporate the host immune system into cancer control started in the 1890s, but these early attempts were disregarded due to inconsistent responses. They were shortly inundated by the development of radiotherapy and chemotherapies, which were found to be more effective [6]. Later, the concept of immunosurveillance emerged in the 1950s, proposing that the immune system, particularly lymphocytes, plays a critical role in controlling the emergence of malignant cells. This idea evolved further when the cancer immune-editing hypothesis was introduced in the 2000s by Schreiber et al., which describes a dynamic and dualistic role of the immune system. According to this model, host immunity acts as an extrinsic tumor suppressor and, paradoxically, as a facilitator of tumor growth and progression through constant interactions between tumor cells, immune cells, and the tumor microenvironment [7]. Consequently, tumor genomics and host immune responses are intricately interconnected and tightly regulated [6].

Drug resistance in cancer therapy is multifaceted and arises from genetic, epigenetic, and microenvironmental factors. A thorough understanding of the molecular mechanisms driving resistance is essential to devise effective strategies to overcome it [8]. Resistance can be broadly classified into intrinsic and acquired phenomena. Intrinsic resistance refers to pre-existing factors within tumor tissues that render them less responsive to treatment even before therapy begins. In contrast, acquired resistance develops during or after treatment, resulting from adaptive cellular changes that diminish the effectiveness of anticancer agents [8]. Emerging approaches, particularly those based on nanotechnology, hold significant promise in overcoming chemoresistance. These strategies enable targeted drug delivery, improving tumor radiosensitization, and remodeling the tumor microenvironments for enhanced therapeutic efficacy [9]. This review thoroughly explores the current understanding of resistance mechanisms to combinations of immunotherapy, radiotherapy, and chemotherapy in locally advanced head and neck squamous cell carcinoma (LA-HNSCC). It emphasizes recent advancements in nanotechnology-driven solutions, metabolic targeting, and dual-target therapies. The goal is to provide insights that can guide the development of next-generation treatment strategies.

## 2 Immunotherapy Combinations in Locally Advanced Head & Neck Squamous Cell Carcinoma (LA-HNSCC)

To date, six phase III trials with published results have evaluated programmed cell death Protein 1 (PD-1)/programmed death-ligand 1 (PD-L1) inhibitors combined with cisplatin-based chemoradiotherapy (CRT) in LA HNSCC, with several additional studies ongoing [4]. In KEYNOTE-412, 804 patients with unresectable stage III–IV disease were randomized to receive either pembrolizumab plus CRT or placebo plus CRT, with event-free survival (EFS) as the primary endpoint [10]. JAVELIN Head and Neck 100 enrolled 907 treatment-naive stage III–IV patients to compare avelumab plus CRT against placebo plus CRT for progression-free survival (PFS) [11]. IMvoke010 randomized 406 high-risk patients following multimodal therapy, with or without primary surgery, to receive adjuvant atezolizumab or placebo for up to one year, with EFS as the primary endpoint [12]. The GORTEC REACH trial stratified 707 patients based on cisplatin eligibility; both cohorts received avelumab, with the eligible arm adding CRT and the ineligible arm pairing cetuximab with radiotherapy, focusing on PFS as the primary endpoint [13]. NIVOPOSTOP (GORTEC 2018-01) randomized 680 high-risk, resected LA HNSCC patients to postoperative CRT with or without adjuvant nivolumab, using disease-free survival (DFS) as its primary endpoint [14]. Finally, KEYNOTE-689 enrolled 714 patients with newly diagnosed resectable stage III–IV LA HNSCC to receive pembrolizumab at all treatment stages, as neoadjuvant therapy, concurrently with radiotherapy with or without cisplatin, and as adjuvant therapy for up to one year, versus standard-of-care (SOC) surgery and adjuvant CRT [15]. The primary endpoint was EFS [15].

While cisplatin-based CRT remains the backbone of curative treatment for locally advanced HNSCC, the addition of PD-1/PD-L1 inhibitors has largely yielded underwhelming results [4]. Although the KEYNOTE-412 trial showed a favorable trend toward pembrolizumab plus CRT compared to placebo, it failed to meet its primary EFS endpoint [10]. Similarly, the JAVELIN Head and Neck 100 trial showed no improvement in PFS or overall survival (OS) with avelumab added to CRT [11]. IMvoke010 also demonstrated no EFS benefit with adjuvant atezolizumab versus placebo [12]. The GORTEC REACH trial suggested improved distant metastasis rates in cisplatin-ineligible cohorts treated with avelumab plus cetuximab and radiotherapy. Still, no significant differences in PFS and locoregional control were observed [13]. Conversely, NIVOPOSTOP (GORTEC 2018-01) reported a significant disease-free survival advantage in PD-L1-positive patients receiving adjuvant nivolumab, marking the first new treatment to show benefit over standard of care (SOC) CRT in resected high-risk LA HNSCC in over two decades [14]. However, OS data are still maturing [14]. KEYNOTE 689 achieved significant improvements in EFS and major pathological response rate (mPR) with pembrolizumab administered across all stages of treatment, leading to the FDA approval on 12 June 2025, for pembrolizumab use as neoadjuvant and continued adjuvant in combination with radiotherapy with or without cisplatin in PD-L1 Combined Positive Score (CPS) ≥ 1 resectable LA HNSCC [15,16]. Collectively, these data confirm that although the safety profiles of PD-1/PD-L1 inhibitors combined with CRT are comparable to CRT alone, durable efficacy in LA HNSCC remains elusive. These mixed outcomes underscore the urgency of integrating predictive biomarkers and refining treatment sequencing to unlock the full potential of ICIs in LA-HNSCC.

Several common design features across these trials may have masked true clinical benefit and warrant attention in future studies. Most trials enrolled unselected LA-HNSCC populations without prospective PD-L1 or HPV stratification, except for KEYNOTE 412 stratifying randomization based on these biomarkers, attenuating potential signals of checkpoint efficacy [12,14,15]. Biomarker-based enrollment may produce heterogeneity in CRT protocols, including variable fractionation regimens, cisplatin schedules, and concurrent targeted agents, complicating cross-trial comparisons and establishing consistent control-arm benchmarks [10,11,13]. These studies administered ICIs at different points in treatment, as neoadjuvant therapy [15], concurrently with CRT [10,11,13], or in the adjuvant setting [12,14,15]. However, no direct head-to-head comparisons have been conducted, leaving the optimal sequencing relative to surgery and CRT undefined. Additionally, since follow-up remains immature for OS in multiple studies, endpoints such as EFS and pathological response may not provide a clear picture of the long-term survival benefit [10,12,14,15]. Finally, correlative biomarker analyses were largely exploratory, lacking prespecified thresholds for PD-L1, ctDNA kinetics, or T-cell clonality, limiting insight into potential mechanistic effects regarding any benefits seen [14,15]. Addressing these issues through biomarker-enriched patient enrollment, standardized CRT regimen, comparative sequencing arms, sufficiently powered endpoints including OS, and robust translational cohorts will be crucial to unlocking the full potential of immunotherapy in LA HNSCC. Summary of phase III trials adding PD-1/PD-L1 inhibitors to chemoradiotherapy in LA-HNSCC is provided in Table 1.

**Table 1:** Phase III (and key Phase II) trials of PD-1/PD-L1 inhibitors added to chemoradiotherapy in LA-HNSCC.

Trial	Phase/N	Setting	ICI (Timing)	CRT Backbone	Primary Endpoint	Result + Key Outcome	Notable Comment
KEYNOTE-412 [10]	III/804	Unresectable stage III–IV	Pembrolizumab (concurrent ± neoadj)	CRT with cisplatin	EFS	Trend favors ICI, but did not meet the primary EFS	Favorable safety; underpowered biomarker subgroups
JAVELIN HN100 [11]	III/907	Newly diagnosed stage III–IV	Avelumab (concurrent)	CRT with cisplatin	PFS	No improvement in PFS or OS	Raised concerns re: timing and patient selection
IMvoke010 [12]	III/406	High-risk post-multimodal therapy	Atezolizumab (adjuvant)	Adjuvant CRT or observation	EFS	No EFS benefit	Mature OS pending
NIVOPOSTOP (GORTEC) [14]	III/680	High-risk resected LA-HNSCC	Nivolumab (adjuvant)	Postop CRT ± nivolumab	DFS	DFS benefit in PD-L1 + subgroup	First positive DFS signal post-surgery
KEYNOTE-689 [17]	III/714	Resectable stage III–IV (neoadj→adjuvant)	Pembrolizumab (neoadj + concurrent + adjuvant)	Surgery ± adjuvant CRT	EFS	EFS improved; mPR ↑; FDA approval (June 12, 2025)	Suggests multi-stage ICI exposure may help in selected pts

Note: CRT, Chemoradiotherapy; EFS, Event-Free Survival; ICI, Immune Checkpoint Inhibitor; PFS, Progression-Free Survival; OS, Overall Survival; DFS, Disease-Free Survival; mPR, Major Pathological Response; PD-L1, Programmed Death-Ligand 1; ↑—improved.

## 3 Resistance Mechanisms to Immunotherapy-Radiotherapy/Chemotherapy Combinations

### 3.1 Immunological Mechanisms

In locally advanced head and neck squamous cell carcinoma (LA-HNSCC), the therapeutic efficacy of immune checkpoint inhibitors (ICIs) combined with chemoradiotherapy (CRT) is frequently undermined by immune evasion within the tumor microenvironment (TME) [18]. A central contributor to resistance is the progressive functional exhaustion of intratumoral CD8^+^ T cells, which co-express multiple inhibitory receptors, including PD-1, T cell immunoglobulin and mucin-domain containing-3 (TIM-3), Lymphocyte-activation gene 3 (LAG-3), and Immunoreceptor tyrosine-based inhibitory motif domains (TIGIT). Recent analyses using single-cell RNA sequencing and multiparametric flow cytometry in both HPV-positive and HPV-negative human HNSCC tumors have shown that PD-1 blockade alone often induces compensatory upregulation of these alternative checkpoints, allowing tumor escape despite initial T-cell activation [19].

Moreover, the TME in HNSCC is heavily populated by myeloid-derived suppressor cells (MDSCs) and regulatory T cells (Tregs), which suppress effector T-cell activity through cytokine production (IL-10, TGF-β), arginase-1 activity, and ROS generation. Importantly, CRT itself exacerbates this immunosuppressive milieu by increasing IL-6, IL-10, and stromal senescence, which promote Treg expansion and MDSC persistence [20]. These immunosuppressive cells not only inhibit antigen-specific T-cell responses but also blunt the formation of effective immune memory, limiting durable responses to ICIs. Taken together, these findings highlight the need for combination immunotherapy approaches targeting PD-1 in tandem with secondary checkpoints or depleting suppressive myeloid and Treg populations to restore immune competence within the TME [21].

### 3.2 DNA Damage Response (DDR) and Cell Survival Pathways

**Figure 1:**
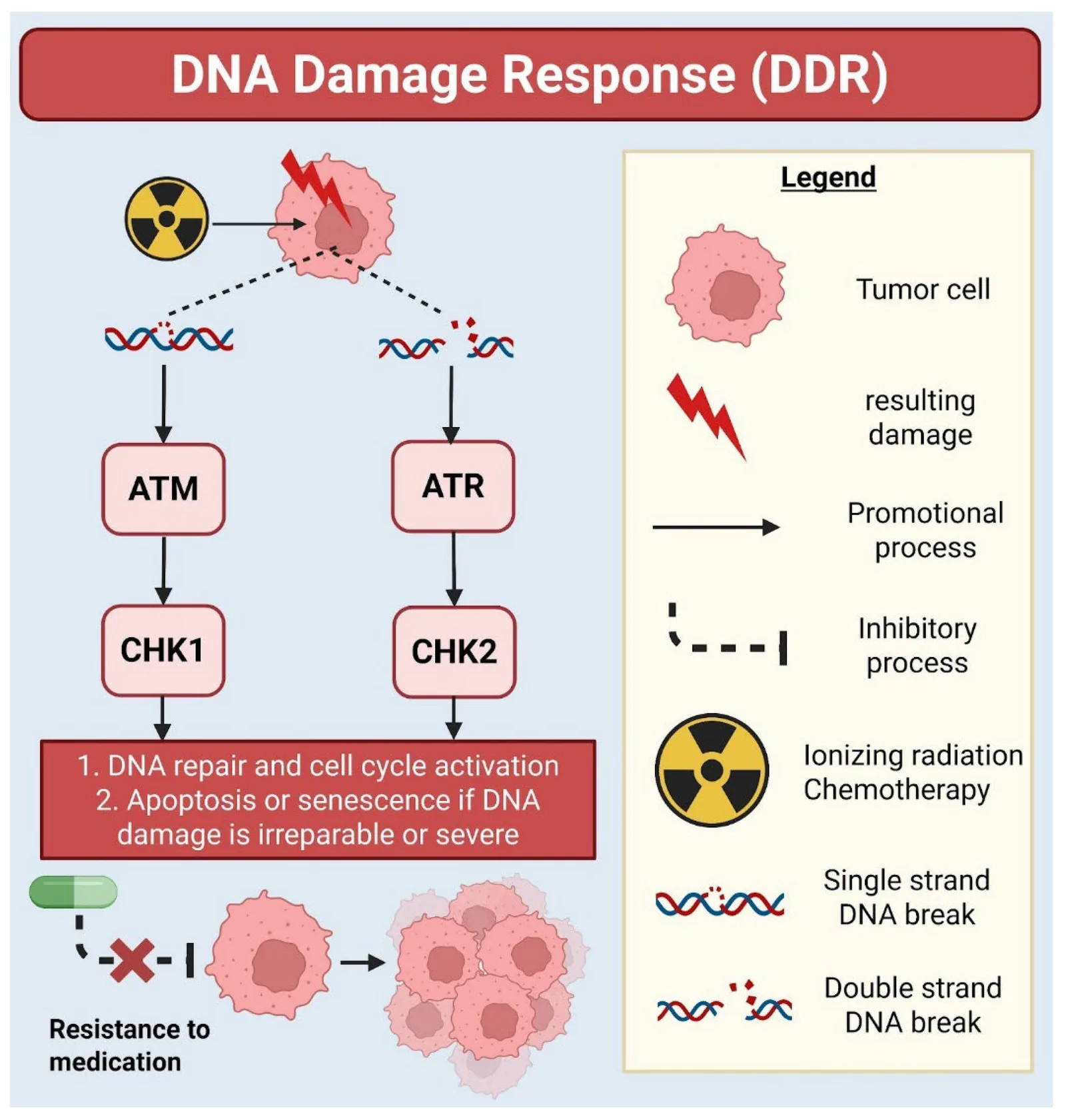
**Simplified diagram of DNA Damage Response (DDR) Pathway.** The DNA damage response (DDR) pathway plays a critical role in determining cancer cell fate following exposure to ionizing radiation or chemotherapy. Such therapies may induce single-strand or double-strand DNA breaks. Damage sensing activates the ATM and ATR pathways, which in turn signal through CHK1 and CHK2 to promote DNA repair and regulate cell-cycle progression. When repair is successful, cancer cells may survive and continue proliferating, potentially contributing to therapeutic resistance in locally advanced HNSCC. Conversely, when DNA damage is extensive or irreparable, cells may undergo apoptosis or senescence. This is a possible explanation for the lack of response, recurrence, or resistance of certain immunomodulatory medications, although more research needs to be done to clarify how targeting DDR can optimize treatment responses in these tumors. ATM, ataxia-telangiectasia mutated; ATR, ataxia-telangiectasia and Rad3-related; CHK1/2, checkpoint kinase 1/2; DDR, DNA damage response; HNSCC, head and neck squamous cell carcinoma. Created in BioRender. Thein, K. (2025) https://BioRender.com/ehd1l3x.

Resistance to CRT and immunotherapy is also driven by tumor-intrinsic defects in DNA damage response (DDR) and cell survival signaling pathways. Critical DDR kinases such as ATM, ATR, and checkpoint kinases CHK1/2 orchestrate cellular responses to double-strand breaks induced by ionizing radiation and platinum-based chemotherapy [22]. However, aberrant activation of these pathways can expedite DNA repair and minimize the formation of cytosolic micronuclei, thereby impairing the cGAS-STING pathway and suppressing type I interferon responses that are essential for immunogenic cell death (ICD) and antigen presentation. In preclinical HNSCC models, ATR or ATM inhibition during RT restored IFN-β secretion and upregulated antigen-processing machinery, enhancing subsequent response to PD-1 blockade [23] (See Fig. 1).

In addition, mutations in TP53, particularly truncating variants common in HPV-negative HNSCC, confer enhanced resistance to CRT by impairing apoptosis and promoting nucleotide excision repair (NER). For example, the TP53-Q331* mutations activate ID2-dependent NER signaling, leading to cisplatin resistance in tongue squamous cell carcinoma [24]. Overexpression of poly (ADP-ribose) polymerase-1 (PARP-1), another DDR effector, facilitates repair of single-strand breaks and reduces CRT-induced cytotoxicity [24]. Notably, PARP inhibition combined with CRT increases calreticulin exposure and dendritic cell activation, synergizing with ICIs in HNSCC xenografts. These mechanistic insights suggest that pairing CRT with targeted DDR inhibition, particularly PARP, ATR, or CHK1 inhibitors, may resensitize tumors to immunotherapy by restoring ICD and innate immune activation via the STING pathway [25]. 

### 3.3 Tumor Hypoxia and Radioresistance

Hypoxia is a defining feature of HNSCC and plays a pivotal role in driving resistance to both radiotherapy and immunotherapy. Under low-oxygen environments, the stabilization of hypoxia-inducible factors-1α (HIF-1α) and hypoxia-inducible factors-2α (HIF-2α) activates the transcription of genes involved in angiogenesis (VEGF), immune escape (PD-L1), and altered cellular metabolism [26]. Elevated HIF expression in hypoxic tumor subregions has been linked to reduced infiltration of CD8^+^ T cells and poor event-free survival. The resulting vasculature is structurally disorganized and highly permeable, creating poorly perfused tumor areas that limit immune cell access and foster a profoundly immunosuppressive microenvironment dominated by MDSCs and Tregs [27].

From a radiobiological perspective, hypoxia markedly diminishes the oxygen enhancement ratio (OER), thereby impairing the production of DNA-damaging free radicals essential for effective radiotherapy. Emerging evidence suggests that ultra-high-dose-rate (UHDR) radiotherapy can partially counteract this limitation [28]. Ultra-high dose rate radiotherapy (FLASH-RT) has been shown to overcome radioresistance and may modulate the tumor microenvironment, with emerging evidence suggesting potential effects on tumor oxygenation and anti-tumor immune responses in head and neck cancer models [28,29]. Furthermore, a validated 15-gene hypoxia signature has been shown to identify patients at higher risk of poor outcomes following CRT combined with immunotherapy, highlighting the importance of incorporating hypoxia-targeting radiosensitizers and vascular normalization strategies to optimize individualized treatment [29].

### 3.4 Non-Immune Microenvironmental Resistance Mechanisms

#### 3.4.1 Cancer-Associated Fibroblasts (CAFs) and Extracellular Matrix (ECM) Barriers

Cancer-associated fibroblasts (CAFs) are one of the most prevalent stromal cell populations within the tumor microenvironment (TME) of locally advanced head and neck squamous cell carcinomas, where they play a crucial role in promoting therapeutic resistance [20,30]. CAFs facilitate immune evasion by secreting immunosuppressive cytokines, including transforming growth factor-β (TGF-β) and interleukin-6 (IL-6), which suppress cytotoxic CD8^+^ T cell activity and promote regulatory T-cell differentiation [20,31]. In addition, elevated levels of CXCL2 further establish a chemokine gradient that sequesters T cells in the stromal compartments and prevents their migration into tumor nests, thereby contributing to an immune-excluded phenotype [32]. Additionally, TGF-β signaling in CAF-rich tumors has been directly associated with resistance to PD-1/PD-L1 blockade, primarily by physically restricting T cells from tumor cores [32].

Furthermore, beyond mediating soluble signals within tumors, CAFs actively reshape the ECM by depositing dense networks of collagen fibers, fibronectin, and hyaluronan, leading to increased matrix stiffness and elevated interstitial fluid pressure [30,33]. This desmoplastic architecture acts as a physical barrier that limits immune cell movement, reduces diffusion of chemotherapeutic agents, and significantly hinders nanoparticle penetration [34]. Stiffer ECM has also been shown to suppress immunogenic cell death following radiotherapy by altering mechanotransduction pathways and decreasing dendritic cell activation [33,35]. Recent clinical studies have identified distinct CAF subtypes, including inflammatory CAFs (iCAFs) and myofibroblastic CAFs (myCAFs), with higher CAF abundance strongly correlating with primary ICI resistance and inferior survival outcomes [36].

Therapeutic strategies targeting CAF-mediated resistance are therefore gaining clinical interest. Approaches such as fibroblast activation protein (FAP)–targeted therapies, integrin inhibitors, and TGF-β pathway blockade aim to counteract CAF-driven immune exclusion [36,37]. Additionally, stromal-normalizing agents such as losartan have demonstrated the ability to reduce collagen deposition, decompress tumor vasculature, improve perfusion, and enhance immune infiltration in preclinical models. Overall, integrating CAF-targeted interventions with immunotherapy-CRT regimens may therefore represent a critical strategy to overcome stromal-driven resistance in LA-HNSCC.

#### 3.4.2 Exosomal Immune Evasion

Tumor-produced exosomes serve as a significant, systemic means of immune evasion, playing a key role in resistance to immunotherapy-based treatments in LA-HNSCC [38]. These nanoscale extracellular vesicles carry a broad array of immunosuppressive proteins, lipids, and nucleic acids that reprogram immune cells locally within the TME and distantly within lymphoid organs. HNSCC-derived exosomes are particularly enriched in PD-L1, Fas ligand (FasL), TGF-β, and regulatory microRNA, factors that work collectively to suppress antigen presentation, trigger T-cell apoptosis, and impair effector T-cell activation [38]. In contrast to membrane-bound PD-L1 on tumor cells, exosomal PD-L1 circulates throughout the body and can suppress T-cell function independently of direct tumor–immune contact, thereby contributing to primary and acquired resistance to PD-1/PD-L1 blockade [39].

In HPV-related HNSCC, exosomal immune evasion is further intensified by the presence of viral oncoproteins (E6 and E7), which alter host gene transcription and immune signaling pathways, primarily by inactivating tumor suppressors such as p53 and pRb [40]. Recent clinical evidence indicates that elevated circulating exosomal PD-L1 levels are a more dynamic and reliable metric for disease activity, correlating strongly with poor response to immune checkpoint inhibitors and inferior survival, often surpassing tissue-based PD-L1 expression as a predictive biomarker. Notably, exosomal PD-L1 levels dynamically change during treatment, suggesting a role in real-time monitoring of therapeutic resistance [40].

Targeting exosome-mediated resistance is an emerging therapeutic strategy. Preclinical approaches include inhibition of exosome production or release using neutral sphingomyelinase inhibitors, prevention of exosome uptake by immune cells, and selective depletion of PD-L1-positive exosomes. Additionally, analyzing the contents of exosomes—such as PD-L1 and immunosuppressive microRNAs—offers a promising, minimally invasive biomarker strategy for patient stratification and early identification of immunotherapy resistance in LA-HNSCC [39].

#### 3.4.3 Microbiome Contributions to Resistance

The oral and gut microbiome have emerged as critical host-level determinants of response and resistance to immunotherapy-based regimens in head and neck squamous cell carcinoma [41]. Commensal microbial communities regulate systemic immune tone by shaping antigen-presenting cell maturation, influencing T-cell priming, and modulating cytokine production. In LA-HNSCC, chemoradiotherapy frequently disrupts microbial diversity through mucosal injury, antibiotic exposure, and nutritional compromise, leading to dysbiosis that undermines effective anti-tumor immunity [42]. Loss of beneficial bacterial taxa—particularly Ruminococcus, Faecalibacterium, and Akkermansia species—has been associated with impaired CD8^+^ T-cell infiltration, reduced interferon-γ signaling, and inferior responses to immune checkpoint inhibitors [43].

The oral microbiome plays an especially important role in HNSCC due to direct tumor–microbe interactions. Pathogenic oral dysbiosis (e.g., Porphyromonas gingivalis and Fusobacterium nucleatum) promotes chronic inflammation, myeloid-derived suppressor cell recruitment, and immune tolerance, while beneficial commensals enhance antigen presentation and cytotoxic T-cell activation [41]. Emerging evidence suggests that microbiome composition influences not only immunotherapy response but also radiotherapy efficacy, as microbial metabolites regulate oxidative stress and DNA damage responses within the tumor microenvironment [44].

These findings have prompted clinical efforts to therapeutically modulate the microbiome. Ongoing trials, including fecal microbiota transplantation (FMT) strategies such as the MARVIN-HN trial, are evaluating whether restoring microbial diversity can enhance the efficacy of immunotherapy in LA-HNSCC [42,45]. Future studies are also exploring probiotics, dietary interventions, and antibiotic stewardship as adjunctive strategies. Incorporating microbiome diversity metrics into clinical trial stratification may therefore improve patient selection and help overcome host-mediated resistance to immuno-CRT combinations.

**Figure 2:**
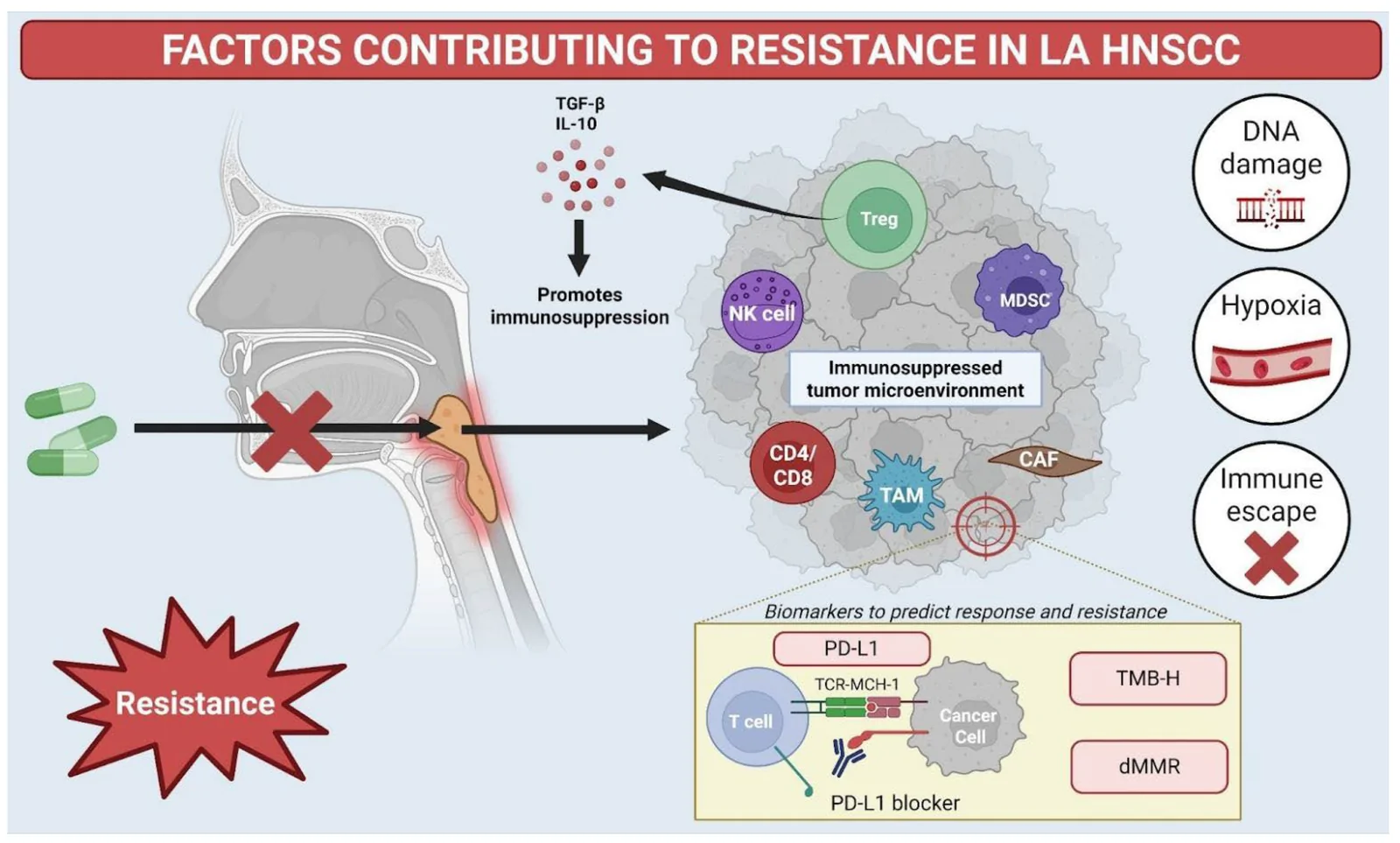
**Factors contributing to resistance in locally advanced (LA) head and neck squamous cell carcinoma (HNSCC)**. Immune checkpoint inhibitors (ICIs) are often ineffective in locally advanced head and neck squamous cell carcinoma (LA-HNSCC) due to multiple resistance mechanisms. A key driver is remodeling of the tumor immune microenvironment (TIME), which consists of cancer cells, CD4^+^ and CD8^+^ T cells, regulatory T cells (Tregs), natural killer (NK) cells, myeloid-derived suppressor cells (MDSCs), cancer-associated fibroblasts (CAFs), tumor-associated macrophages (TAMs), and other stromal or immune populations. Immunosuppressive cytokines such as TGF-β and IL-10 further dampen anti-tumor immunity. Additional resistance mechanisms include DNA damage, tumor hypoxia, and immune escape pathways. Predictive biomarkers, including PD-L1 expression, tumor mutational burden-high (TMB-H), and mismatch repair deficiency (dMMR), may provide insight into the likelihood of response or resistance in these cancers. Created in BioRender. Thein, K. (2025) https://BioRender.com/ehd1l3x.

A summary on resistance mechanisms to ICI + CRT in LA-HNSCC and actionable interventions (including nanotech & biomarkers) is provided in Table 2. Refer to Fig. 2 for a graphical summary of factors contributing to resistance in locally advanced (LA) head and neck squamous cell carcinoma (HNSCC).

**Table 2:** Resistance mechanisms to ICI + CRT in LA-HNSCC and actionable interventions (including nanotech & biomarkers).

Resistance Mechanism	Biological Basis/Markers	Evidence (HNSCC/Preclinical)	Targeted Interventions
T-cell exhaustion/compensatory checkpoints	PD-1, TIM-3, LAG-3, TIGIT upregulation; exhausted CD8 T cell signatures	scRNA-seq shows compensatory checkpoint upregulation. after PD-1 blockade	Dual/quad checkpoint blockade (anti-PD-1 + anti-TIM-3/TIGIT/LAG-3); adoptive CAR approaches
DDR activation/rapid DNA repair	ATM/ATR/CHK1/2 activity; PARP-1 expression; TP53 mutations	ATR/ATM inhibition improves RT-induced IFN signaling preclinically	PARP/ATR/CHK1 inhibitors ± CRT + ICI; nanoparticle delivery of DDR inhibitors to tumor to limit toxicity
Tumor hypoxia/radioresistance Hypoxia-targeted radiosensitizers, vascular normalization (anti-VEGF)	HIF-1α/HIF-2α, hypoxia gene signature, low perfusion by PET	Hypoxia signatures predict poor CRT+ICI outcomes; UHDR-RT preclinical	oxygen carriers; Au NPs or gadolinium NPs as radiosensitizers
CAF/ECM immune exclusion	CAF markers (FAP, α-SMA), dense collagen, CXCL12 levels	CAF-rich tumors exclude CD8 T cells and predict anti-PD-1 failure	FAP-targeted agents, integrin inhibitors, losartan preconditioning; collagen-degrading nanoparticle co-therapies
Metabolic competition (glycolysis, lactate)	High LDH-A, MCT1/4, lactate signature; glutaminase activity; IDO1	Glycolytic & lactate signatures link to low CD8 and poor EFS; IDO1 trials mixed	LDH-A inhibitors, MCT inhibitors, glutaminase inhibitors (telaglenastat), adenosine A2A/A2B antagonists; nanoparticle delivery to the tumor to reduce systemic toxicity
HER-family bypass (EGFR → HER3/HER2)	HER3 upregulation, heregulin (HRG) expression, HER2 amplification	Cetuximab resistance associated with HER3 activation; preclinical	rescue with dual blockade Pan-HER TKIs (afatinib), anti-HER3 antibodies (duligotuzumab), combinations with ICI
Exosomal and microbiome-mediated suppression	Exosomal PD-L1, miR-21; loss of beneficial gut/oral commensals	Exosomal PD-L1 predicts poor ICI response; microbiome modulates ICI efficacy	Exosome depletion/uptake inhibitors, FMT or microbiome modulation; local nanoparticle vaccines to alter APC priming

Note: MDSCs, Myeloid-Derived Suppressor Cells; ATM, ataxia-telangiectasia mutated; ATR, ataxia-telangiectasia and Rad3-related; CHK1/2, checkpoint kinase 1/2; PARP-1, Poly (ADP-ribose) polymerase; STING pathway, Stimulator of Interferon Genes pathway, HIF-1α/HIF-2α, Hypoxia-Inducible Factors; MCT1/4, Monocarboxylate Transporters; FAP, Fibroblast Activation Protein; ID1/IDO1, Indoleamine 2,3-Dioxygenase 1; A2A/A2B, adenosine receptors; ctDNA, Circulating Tumor DNA.

### 3.5 Integrative Conceptual Framework of Resistance in LA-HNSCC

While mechanisms underlying resistance to immune checkpoint inhibitors (ICIs) and chemoradiotherapy (CRT) have traditionally been considered as separate entities, recent studies indicate that these mechanisms are part of a dynamic, interconnected, and partially hierarchical network rather than functioning independently [6]. The current hypothesis is that in locally advanced HNSCC (LA-HNSCC), resistance emerges from a complex, multi-layered system composed of upstream environmental factors, stromal influences, and downstream adaptations within immune and tumor cells [46]. At the top of this hierarchy, tumor hypoxia and metabolic reprogramming act as key upstream regulators. Hypoxia leads to the stabilization of HIF-1a and HIF-2a, which drive PD-L1 expression, enhance glycolysis, elevate lactate production, and promote VEGF-mediated abnormal angiogenesis [47]. These changes collectively decrease the effectiveness of radiotherapy by reducing the oxygen enhancement effect and hindering immune cell infiltration, resulting in resistance to both CRT and ICIs. Likewise, metabolic competition for crucial nutrients such as glucose, glutamine, and tryptophan produces a nutrient-poor microenvironment, impairing T-cell function and supporting tumor survival after DNA damage [46].

At the intermediate level, cancer-associated fibroblasts (CAFs) and remodeling of the extracellular matrix (ECM) serve as structural enhancers of resistance [48]. The stiffening of the stroma and increased collagen deposition impair drug distribution, limit nanoparticle penetration, and restrict immune cell movement, thereby reducing both the cytotoxic effects of CRT and the immune activation induced by ICIs [35]. Exosomal communication further propagates resistance on a systemic scale, enabling tumors to inhibit dendritic cell maturation and T-cell activation beyond the primary tumor environment [48]. On the downstream end, tumor-intrinsic changes, such as upregulated DNA damage response (DDR) pathways and the compensatory expression of immune checkpoints, further promote therapeutic resistance [49]. Enhanced DNA repair mechanisms decrease immunogenic cell death and limit the presence of cytosolic DNA, thereby suppressing cGAS-STING pathway activation and antigen presentation [23]. 

Notably, resistance to CRT and ICIs is biologically interdependent rather than a separate phenomenon. In principle, effective CRT may enhance immunogenic cell death, promote neoantigen release, and activate STING-dependent interferon signaling, thereby increasing tumor sensitivity to ICIs. Nevertheless, several pivotal Phase III trials—such as KEYNOTE-412 [10], JAVELIN Head & Neck 100 [11], and the Phase II/III NRG-HN004 trial [50]—have failed to demonstrate this synergy in unselected patient populations. These negative outcomes underscore the uncertainty regarding whether resistance mechanisms function in parallel, sequentially, or within a hierarchical network led by upstream factors. In NRG-HN004, substituting cetuximab with durvalumab for cisplatin-ineligible patients did not improve clinical outcomes and even trended toward poorer locoregional control, suggesting that PD-L1 inhibition alone may not be sufficient to surmount the inherent resistance of the HNSCC tumor microenvironment [50]. When severe hypoxia or heightened DDR activation occurs, CRT cannot adequately prime the immune system, imposing a “ceiling effect” on ICI efficacy. Conversely, existing immune exclusion or stromal barriers mediated by CAFs may limit the translation of CRT-induced immune responses into lasting systemic effects. Therefore, resistance in LA-HNSCC is best viewed as a systems-level network in which upstream metabolic stress governs the structural and immunological landscape of the tumor. Understanding this complexity implies that future therapeutic advances may depend on combination strategies that target hypoxia, stromal remodeling, and DDR pathways simultaneously, rather than addressing each mechanism in isolation.

### 3.6 Translating Resistance Mechanisms into Targeted Therapeutic Strategies

The resistance mechanisms described above not only illuminate the biological complexity of locally advanced head and neck squamous cell carcinoma (LA-HNSCC) but also identify multiple actionable therapeutic vulnerabilities that may be exploited to improve the efficacy of immunotherapy-based treatment combinations. Increasingly, translational research has focused on targeting the specific biological pathways that drive resistance to chemoradiotherapy (CRT) and immune checkpoint inhibitors (ICIs), thereby converting mechanistic insights into rational therapeutic strategies.

One primary axis of resistance involves the dysregulation of DNA damage response (DDR) pathways. Tumor cells subjected to radiotherapy and platinum-based chemotherapy often co-opt DDR signaling networks—specifically the ATR, ATM, and CHK1/2 pathways—to repair genomic insults and evade immunogenic cell death [49]. This robust adaptive repair capacity limits the release of tumor-associated antigens and damage-associated molecular patterns (DAMPs) that are necessary for robust immune priming [49]. Pharmacologic inhibition of DDR kinases through agents such as ATR, CHK1, or PARP inhibitors has emerged as a potent strategy to sensitize tumors to CRT while simultaneously augmenting their immunogenicity. By disrupting efficient DNA repair, these inhibitors facilitate the accumulation of cytosolic DNA, thereby activating the cGAS–STING innate immune sensing pathway, which promotes dendritic cell maturation and enhances T-cell recruitment [51].

Chronic intratumoral hypoxia represents another formidable barrier, particularly in the bulky, poorly vascularized tumors characteristic of LA-HNSCC [52]. Hypoxic signaling not only confers radioresistance but also orchestrates an immunosuppressive microenvironment by upregulating vascular endothelial growth factor (VEGF), adenosine-generating enzymes (CD39/CD73), and inhibitory checkpoint ligand [47]. Strategies designed to alleviate or exploit hypoxia—including hypoxia-activated prodrugs, HIF-1α inhibitors, and anti-angiogenic agents—are currently being investigated to restore the intratumoral oxygen tension required for both radiation efficacy and T-cell function. However, despite more than 40 years of work, there are no HAPs approved by the FDA [52]. By mitigating hypoxia-driven immune exclusion, these approaches may broaden the therapeutic window for ICIs and facilitate more durable clinical responses. Similarly, preclinical studies have shown that strategies targeting the TGF-beta pathway or using extracellular matrix (ECM)-modifying agents can normalize the tumor stroma, leading to improved immune cell migration and enhanced immune checkpoint inhibitor (ICI) activity. By addressing resistance mediated by cancer-associated fibroblasts (CAFs), these interventions aim to transform ‘cold’ or immune-excluded tumors into ‘hot’, immune-sensitive environments with stronger anti-tumor responses. However, the overall success of these approaches has been limited, underscoring the need for further research into the complex interactions among the ECM, cancer cells, and stromal cells to identify novel therapeutic targets and strategies [53]. Therapeutic interventions targeting metabolic checkpoints—such as IDO1 inhibitors, including Epacadostat—are also being developed to restore immune competence; however, no objective response has been observed in a single-agent trial to date in any malignancy [54]. By alleviating metabolic competition, these strategies provide a more hospitable environment for T-cell activation and survival during combination therapy.

Collectively, these advancements illustrate a paradigm shift toward mechanism-informed combination therapies. Rather than empirically adding novel agents, targeting the fundamental biological processes of DNA repair, hypoxia signaling, stromal exclusion, and metabolic suppression offers a rational framework for overcoming resistance. These mechanism-guided interventions, summarized in Table 2, represent the next frontier in translating the molecular landscape of LA-HNSCC into improved survival outcomes for patients undergoing immunotherapy-based treatment.

## 4 Metabolic Adaptations Driving Resistance to Immunotherapy Combinations

### 4.1 Tumor Cell Metabolism and Immune Evasion

Head-and-neck squamous-cell carcinomas (HNSCC) exhibit a highly glycolytic phenotype even under normoxia, thereby maintaining a steep glucose gradient that deprives tumor-infiltrating lymphocytes (TILs) of fuel while lowering intra-tumoral pH. In a multi-omics analysis of 510 LA-HNSCC samples, high expression of a 233-gene lactate-metabolism signature was independently associated with reduced CD8^+^ T-cell density, elevated T-cell exhaustion markers, and worse event-free survival, underscoring glycolysis as a driver of immune escape [55]. 

Mechanistically, aerobic glycolysis promotes immune evasion by upregulating PD-L1 expression through hypoxia-inducible factor-1α (HIF-1α) signaling and impairing TNF-α-mediated cytotoxicity, as demonstrated in CRISPR-based studies across solid tumors, including HNSCC [56]. In addition, glutamine metabolism also provides a second metabolic shield. Isotope-tracing studies demonstrate that glutaminolysis sustains tumor redox balance and supplies α-ketoglutarate for epigenetic remodeling that reinforces T-cell exclusion. In pre-clinical HNSCC xenografts, pharmacologic glutaminase inhibition with telaglenastat (CB-839) reduced intratumoral ROS buffering, restored dendritic-cell activation, and sensitized tumors to radiation and anti-PD1 therapy [57].

Competition for essential amino acids further suppresses effector immunity. Up-regulation of indoleamine-2,3-dioxygenase 1 (IDO1) depletes tryptophan and generates the immunosuppressive metabolite kynurenine, driving expansion of FOXP3^+^ regulatory T cells and AHR-dependent T-cell dysfunction [58]. IDO1 overexpression contributes to immunosuppressive remodeling of the tumor microenvironment. It is associated with poor prognosis across multiple solid tumors, including HNSCC [58]. Pharmacodynamic biopsies from a neoadjuvant nivolumab ± BMS-986205 window-of-opportunity trial confirmed rapid kynurenine/tryptophan ratio normalization only in responders [59]. However, current NCCN guidelines consider IDO1 a prognostic rather than predictive biomarker and do not recommend its use for treatment selection outside clinical trials.

Lactate accumulation resulting from enhanced tumor glycolysis contributes to acidification of the tumor microenvironment and promotes immunosuppressive signaling, including macrophage polarization toward a pro-tumorigenic phenotype [55,56]. In a Journal of Clinical Investigation study, LDH-A inhibition redirected glucose metabolism toward the pentose phosphate pathway, reduced extracellular lactate levels, and enhanced CD8^+^ T-cell proliferation, converting anti–PD-1–resistant tumors into responders [60]. 

**Figure 3:**
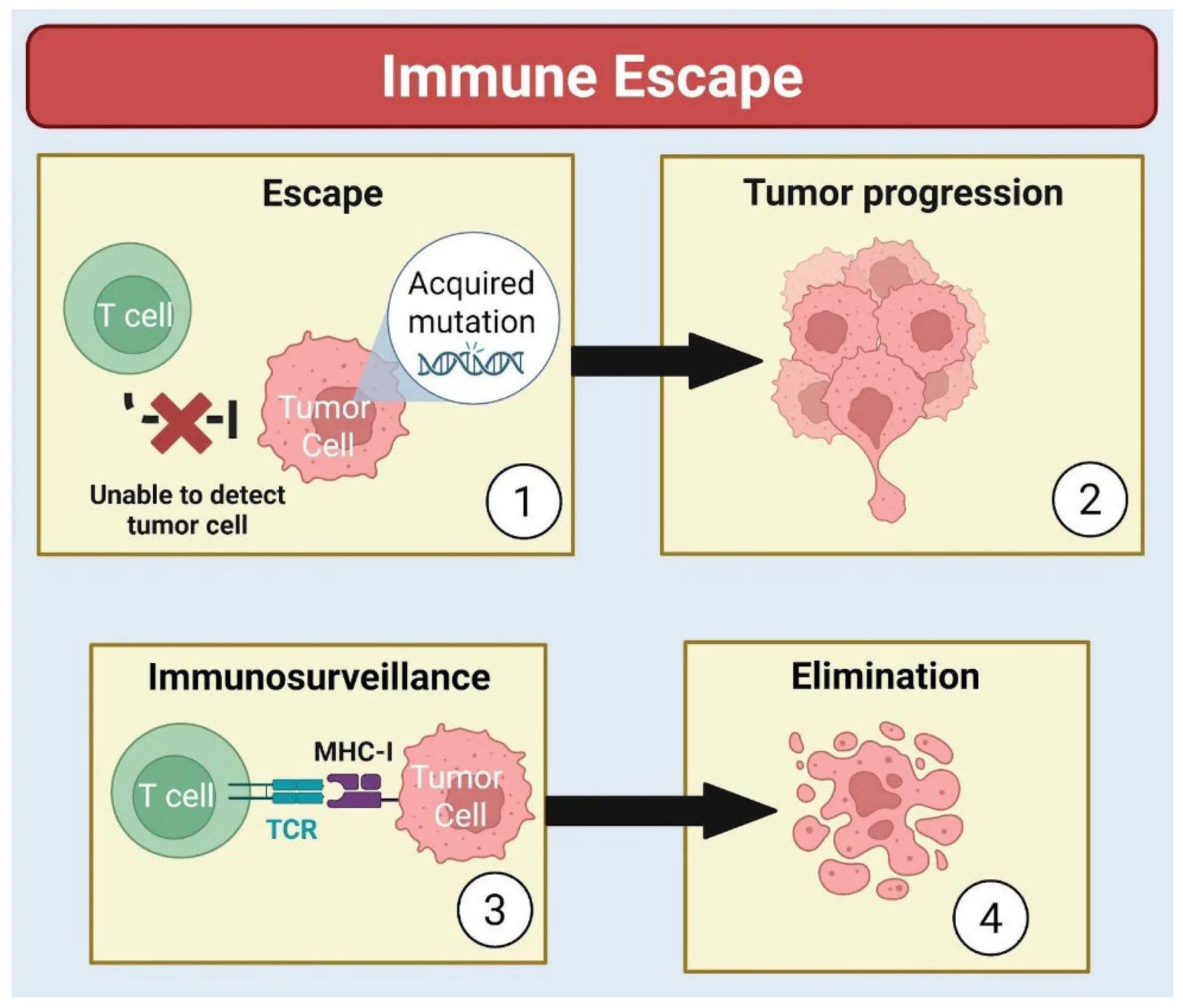
**Basic mechanism of immune escape**. (1) Escape: Tumor cells may acquire mutations or phenotypic changes that allow them to evade recognition by T cells and other immune cells. (2) Tumor progression: In the absence of effective immune clearance, tumor cells proliferate and spread. (3) Immunosurveillance: T cells can sometimes recognize and respond to tumor antigens via T Cell Receptor (TCR)—Major Histocompatibility Complex class I (MHC1) interaction. (4) Elimination: Effective immune recognition and activation lead to the destruction of tumor cells, representing the desired anti-tumor immune response. Created in BioRender. Thein, K. (2025) https://BioRender.com/ehd1l3x.

Finally, adenosine generated by ectonucleotidases CD39/CD73 accumulates in hypoxic niches. Binding to A2A and A2B receptors on immune cells suppresses IL-2 and IFN-γ production, limits dendritic-cell IL-12 secretion, and fosters Treg expansion. A 2025 pan-cancer review reported that A2A signaling genes are among the top up-regulated checkpoints in HNSCC and correlate inversely with cytolytic-score signatures [61]. Collectively, these interconnected metabolic alterations reshape the tumor microenvironment and limit the efficacy of PD-1/PD-L1 blockade and CRT-based combination therapies in LA-HNSCC. Fig. 3 provides a simplified illustration of the basic mechanism of immune escape.

### 4.2 Targeting Metabolic Pathways to Reverse Resistance

#### 4.2.1 IDO1 and Tryptophan Catabolism

The combination of the IDO1 inhibitor epacadostat plus pembrolizumab failed to improve overall survival in the phase III KEYNOTE-669/ECHO-304 trial in recurrent/metastatic HNSCC, highlighting the limitations of IDO1 monotherapy [62]. However, a response-adaptive neoadjuvant trial (NCT03854032) combining nivolumab with the potent IDO1 inhibitor BMS-986205 achieved a major pathologic response (≤10% viable tumor) in 38% of patients compared to 18% with nivolumab alone, alongside marked expansion of tumor-resident clonally-expanded CD8^+^ T cells [59]. These findings suggest that complete pathway inhibition and appropriate treatment timing may be critical.

#### 4.2.2 Glutaminase Inhibition

Telaglenastat demonstrated radiosensitization in HNSCC xenografts and reduced clonogenic survival in patient-derived organoids, permitting >50% reduction in cisplatin dose without loss of tumor control [57]. In an early phase I monotherapy clinical study (NCT02071862), telaglenastat exhibited disease control rates of approximately 43–50% across various solid tumors; it did not include combination with nivolumab [63]. In a separate phase I/II trial [64], telaglenastat plus nivolumab showed modest activity in melanoma, renal cell carcinoma, and NSCLC (ORR 8.4%), without HNSCC-specific durable responses. Ongoing LA-HNSCC trials are now integrating telaglenastat with concurrent chemoradiotherapy and pembrolizumab to potentiate immunogenic cell death and overcome resistance. 

#### 4.2.3 Adenosine-Axis Blockade

First-generation selective A2A antagonists, such as ciforadenant, have demonstrated restoration of T-cell cytokine production but a limited single-agent activity; therefore, a dual A2A/A2B antagonism may be required for a significant clinical benefit. First-generation selective A2A antagonists, such as ciforadenant, restored T-cell cytokine production and showed modest activity in treatment-refractory renal cell carcinoma models [65]. Preclinical efforts in HNSCC now explore dual A2A/A2B blockade, including agents like M1069, to overcome adenosine-mediated suppression, although peer-reviewed data in this context remain limited.

#### 4.2.4 Lactate/LDH Targeting

LDH-A inhibitors such as FX11 or stiripentol reduce lactate export, normalize tumor pH, and relieve A2A-mediated T-cell anergy [64]. Verma et al. demonstrated that LDH inhibition redirects glucose to T cells and renders tumors susceptible to CTLA-4 or PD-1 blockade, achieving complete regression in 60% of mice versus 10% with immunotherapy alone [60].

#### 4.2.5 Emerging Combinations and Challenges

Multi-arm adaptive platforms are now testing “vertical” combinations (e.g., IDO1 + A2A blockade) to forestall metabolic escape routes, while circulating metabolites (kynurenine, lactate) and hyperpolarized ^13^C-MRI are being explored as on-treatment pharmacodynamic biomarkers [54,61]. Limitations include overlapping on-target toxicities, particularly neurocognitive effects with A2A inhibition and mitochondrial stress with glutaminase blockade, as well as metabolic plasticity that allows tumors to switch to fatty-acid oxidation. Integrated biomarker-driven selection, real-time metabolic imaging, and rational sequencing with radiotherapy are therefore essential to convert transient metabolic relief into durable clinical benefit for patients with LA-HNSCC.

## 5 Nanotechnology—Driven Strategies to Overcome Immunotherapy Resistance

### 5.1 Nanomedicine: Principles and Current Landscape

Despite advances in treatments and the emergence of immune checkpoint inhibitors, HNSCC remains a prevalent malignancy globally, with low survival rates due to limitations in bioavailability and poor targeting of treatment options, reducing efficacy [66]. As such, nanotherapy has emerged as a promising strategy to be added to the therapeutic regimen as it offers several unique benefits, such as precise drug delivery, augmenting drug delivery, enhanced drug stability, and reduced systemic toxicity owing to favorable solubility and biocompatibility profiles [66]. 

Nanoparticles (NP) are manufactured in a variety of sizes ranging from 1 to 100 nm and are engineered in a variety of types, including metal, metal oxide, mesoporous, polymer, liposomal, and others, each conferring differing unique attributes such as strong electrical conductivity, optical absorption, and unique fluorescent properties [66]. Delivery of these nanocarriers and NPs into the cytosol of the tumor cell and TME requires a complex, intricate five-step process referred to as the CAPIR cascade [67]. The CAPIR cascade consists of: (1) circulation within blood vessels, (2) accumulation at the site of the tumor, (3) penetration into the tumor, (4) internalization by tumor cells, and (5) drug release [68]. A study utilizing a high-throughput lipid nanoparticle screening assay was employed to assess the *in vivo* nucleic acid delivery efficiency of 94 chemically unique nanoparticles to HNSCC solid tumors. Through DNA barcoding, a novel lipid nanoparticle was identified for its ability to precisely target HNSCC tumors following systemic administration preferentially. Notably, the nanoparticle continued to selectively accumulate in HNSCC tissues with reduced off-target delivery to the liver [69].

### 5.2 Nanotechnology-Based Immune Modulation

One of the biggest obstacles in malignancy treatment is multidrug resistance, which is in part due to the tumor microenvironment (TME) [67]. The TME consists of a variety of different cell types, with the most notable being immune cells, stromal cells, vessels, and the extracellular matrix. The interaction between tumor cells and the TME promotes both tumor progression and multidrug resistance [67]. Nanomedicine has shown promising results in reshaping the TME via suppressing fibroblasts, promoting M1 macrophage polarization, aiding dendritic cell maturation, and encouraging T cell infiltrations. All these modifications of the TME enhance immunotherapy and immune modulation, conferring improved efficacy [70].

Tumor vaccines, such as the HPV E6 and E7 vaccine, contain tumor antigens that activate immune cells to induce a robust immune response. These vaccines have substantial efficacy and a favorable safety profile; however, the immunosuppressive environment within the TME substantially inhibits the efficacy of tumor vaccines in HNSCC [66]. To overcome this, Nanomedicine has also been utilized alongside tumor vaccines as it augments their efficacy and counteracts suppression, in part due to the TME remodeling mentioned above [66].

### 5.3 Nanoparticle-Based Radiosensitization and Chemosensitization

In general, radiotherapy is typically the most straightforward treatment modality for solid tumors; however, especially in high doses, there is considerable collateral damage to proximal, healthy tissue [71]. As such, radiosensitizers have emerged as an effective method to boost the efficacy of radiotherapy while also mitigating damage to normal cells [71]. Among these, certain NPs (gold, gadolinium, ultrasmall clusters) act as radiosensitizers, amplifying DNA damage as they accumulate in tumors. Among metallic radiosensitizers, gold (Au) NPs are especially attractive given their strong interaction with radiation due to Au’s high atomic number, excellent stability, and low toxicity [71]. Indeed, Au can enhance the radiation dose received by the tumor by 200% or higher when compared to tumor tissue not loaded with Au due to the direct interaction between Au and radiation. When the radiation rays directly hit the Au NPs, the NP’s themselves become a new source of radiation as they emit scattered photons and electrons, causing free radical damage via reactive oxygen species to the surrounding tissue, resulting in DNA damage [66,71]. As an alternative to Au, silver has also demonstrated capabilities to absorb, scatter, and emit radiation, enhancing radiotherapy efficacy [66].

Similar to radiotherapy, chemotherapy is also a mainstay of cancer therapeutic regimens; however, can also cause significant damage to healthy cells. Leveraging NPs’ ability to accumulate in tumor tissue preferentially, they can decrease systemic toxicity, thereby making them an ideal candidate to deliver chemosensitizers [72]. A study by Sethi et al. demonstrated that chemosensitizers wortmannin and olaparib formulated with NPs retained strong chemosensitization effects in lung cancer cell lines and multidrug-resistant lines [72]. In addition, the NP formulations led to greater accumulation in tumor tissue compared to free drugs, with significantly less liver and blood toxicity compared to their small-molecule counterparts [72]. Taken together, these studies demonstrate potential benefits in both efficacy and mitigation of adverse effects with radiotherapy and chemotherapy with the use of NPs. 

### 5.4 Emerging Clinical Trials of Nanoparticle-Based Immunotherapy Strategies

Although much of the nanotechnology literature in LA-HNSCC remains preclinical, several nanoparticle-based strategies have advanced into early-phase clinical development, particularly in combination with immunotherapy. Hafnium oxide nanoparticles (NBTXR3), which function as intratumoral radioenhancers, have been evaluated in clinical trials, including NCT04892173, in which they are combined with radiotherapy and pembrolizumab for locally advanced HNSCC, to amplify radiation-induced immunogenic cell death and enhance antitumor immune responses [73]. Earlier studies, including NCT03589339, have also explored NBTXR3 in combination with radiotherapy and anti–PD-1 agents in recurrent and metastatic settings, supporting its role in potentiating immune-mediated tumor control [74]. In parallel, lipid nanoparticle (LNP)-based mRNA vaccines, such as the personalized neoantigen vaccine mRNA-4157 (V940), are being investigated in combination with pembrolizumab (NCT04526899) to enhance tumor-specific T-cell priming and overcome resistance to immune checkpoint inhibition [75]. Similarly, other mRNA-LNP platforms, including FixVac (e.g., BNT111; NCT03480152), are being evaluated in combination with ICIs to improve antigen presentation and immune activation across solid tumors [76]. Nanoparticle-based chemotherapeutic delivery systems, such as albumin-bound paclitaxel (nab-paclitaxel), are also under investigation in combination with pembrolizumab (e.g., NCT03799445), aiming to enhance tumor antigen release while maintaining tolerable systemic toxicity [77]. Additionally, liposomal formulations and nanocarrier-based drug delivery systems, such as liposomal irinotecan combined with nivolumab (NCT04363801), are being explored to improve intratumoral drug accumulation and optimize synergy with immunotherapy [78]. Early-phase trials are also evaluating nanoparticle-mediated delivery of innate immune activators, such as STING agonists (e.g., NCT04096638), which aim to stimulate dendritic cell activation and convert immunologically “cold” tumors into “hot” tumors more responsive to ICIs [79]. Collectively, these emerging clinical studies provide early proof-of-concept that nanoparticle-based platforms can enhance multiple steps of the cancer–immunity cycle; however, their definitive clinical benefit in LA-HNSCC remains to be established.

## 6 Oncogenic ERBB (EGFR/HER2/HER3) Bypass Signaling as a Tumor-Intrinsic Driver of Resistance to Immunotherapy-Based Combinations

In locally advanced head and neck squamous cell carcinoma (LA-HNSCC), combining immunotherapy with radiotherapy or chemotherapy has become an increasingly common approach to potentiate anti-tumor immunity and improve clinical outcomes. Despite these advances, therapeutic resistance continues to be a significant barrier to long-term success. While immune evasion and tumor microenvironment factors play essential roles, growing evidence highlights oncogenic bypass signaling via the HER (ErbB) receptor family—particularly EGFR, HER2, and HER3—as a critical mechanism of resistance across various treatment modalities, including those that incorporate immunotherapy [80].

Cetuximab, a monoclonal antibody targeting EGFR, continues to be a cornerstone of treatment in conjunction with radiotherapy, improving locoregional control and overall survival [81]. Nonetheless, resistance frequently develops, with HER3 emerging as a critical mediator. Preclinical studies have demonstrated that cetuximab treatment induces upregulation of HER3 and promotes HER2/HER3 dimerization, enabling tumor cells to circumvent EGFR blockade [80]. Although HER3 lacks intrinsic kinase activity, it forms heterodimers with EGFR or HER2, triggering downstream PI3K/AKT signaling pathways. Notably, inhibiting HER3 has been shown to restore cetuximab sensitivity, emphasizing its central role in resistance. In HNSCC, the PI3K/AKT/mTOR pathway is a major oncogenic driver, often by activation of HER3-PI3K molecular complexes, even in the absence of PIK3CA mutation [82]. Consequently, elucidating the mechanisms through which the PI3K/AKT axis mediates immune resistance is essential for understanding therapeutic failure and for developing effective combination strategies [82]. Current evidence suggests the suppression of anti-tumor immunity by PI3K/AKT signaling operates via three principal “immunosuppressive pillars”. 

First, oncogenic PI3K signaling actively subverts the IFN-γ/STAT1 pathway, leading to the profound downregulation of MHC Class I and II molecules. Experimental inhibition of PI3K has been shown to restore STAT1 protein stability and enhance genomic accessibility at STAT1 binding motifs, thereby “re-labeling” tumor cells for T-cell recognition; conversely, the loss of PTEN and subsequent AKT activation further facilitates immune escape by reducing surface MHC-I expression [83]. Second, the AKT/mTOR axis establishes an immunologically “cold” tumor microenvironment by directly modulating the tumor secretome, specifically upregulating IL-6 and TGF-beta. While IL-6 serves as a primary recruiter for myeloid-derived suppressor cells (MDSCs) that biochemically dampen T-cell activation, TGF-beta acts as a master regulator of immune exclusion by activating cancer-associated fibroblasts (CAFs) [84]. These CAFs deposit a dense collagenous extracellular matrix that functions as a physical barrier, effectively sequestering CD8^+^ T-cells within the stroma and preventing their infiltration into the tumor parenchyma [84]. Finally, the PI3K/AKT pathway ensures checkpoint-mediated evasion by promoting the expression and post-translational stabilization of PD-L1. This axis not only reshapes the microenvironment to recruit immunosuppressive populations—including regulatory T-cells (Tregs), MDSCs, and M2-polarized macrophages—but also maintains high surface levels of PD-L1 to provide a continuous inhibitory signal to infiltrating lymphocytes [85]. The therapeutic relevance of this link is underscored by evidence that inhibitors targeting JAK/STAT3 (e.g., AG490) or PI3K/AKT (e.g., LY294002) significantly reduce PD-L1 density [85]. Collectively, these mechanisms suggest that HER-family-driven PI3K activation represents a dominant upstream resistance program that must be neutralized to restore the efficacy of immunotherapy in locally advanced disease.

This resistance is further compounded by heregulin, a ligand that activates HER3 signaling. Tumors with high heregulin expression are less responsive to cetuximab but remain sensitive to broader HER-family inhibitors, suggesting that selective EGFR blockade alone is insufficient in the context of ligand-driven HER3 activation [86]. In this setting, HER3 sustains survival signaling despite concurrent radiotherapy, chemotherapy, or immune-based therapies.

HER2 amplification—well characterized in lung adenocarcinoma—may serve as a similar escape mechanism in HNSCC. Amplification of HER2 has been shown to drive resistance to EGFR-TKIs in tumors lacking common secondary EGFR mutations, such as T790M [87]. By activating redundant survival pathways, HER2 enables tumor cells to evade the cytotoxic effects of EGFR inhibition and contributes to resistance across multiple treatment platforms. These converging mechanisms within the HER signaling network ultimately diminish the synergistic potential of immunotherapy combined with radiotherapy or chemotherapy.

### Strategies to Overcome HER-Mediated Resistance in Multimodal Regimens

In response to the limitations associated with EGFR monotherapy, novel strategies targeting multiple members of the HER family have emerged to overcome resistance in immunotherapy, radiotherapy/chemotherapy combinations. One such approach involves pan-HER tyrosine kinase inhibitors (TKIs) such as afatinib, which irreversibly inhibit EGFR, HER2, and HER4. Preclinical studies have demonstrated that afatinib effectively suppresses heregulin-driven HER3 activation and restores sensitivity to EGFR inhibition in both lung and head and neck cancer models [86,87]. Its ability to block multiple HER receptors simultaneously makes it a promising candidate for use in combination with immunotherapy or cytotoxic treatments.

Another promising strategy employs dual-targeting antibodies, such as duligotuzumab, which simultaneously inhibits EGFR and HER3. This dual blockade has been shown to overcome cetuximab resistance and enhance radiosensitivity in HNSCC xenograft models [88]. By disrupting EGFR-HER3 signaling and suppressing downstream PI3K/AKT pathways, duligotuzumab helps restore the efficacy of radiation and chemotherapy. In the context of immunotherapy, this may also enhance immune-mediated killing by reducing tumor cell survival and altering the immune microenvironment.

Collectively, these approaches highlight the potential of HER-targeted intensification strategies to improve the durability of multimodal regimens. Future clinical trials should focus on biomarker-guided patient selection, identifying tumors with high HER3 expression, HER2 amplification, or heregulin production to tailor treatment. Integrating pan-HER or HER3-targeted agents may not only resensitize tumors to radiation and chemotherapy but also potentiate immunotherapy, offering a path forward for patients with resistant disease.

## 7 Biomarkers Predicting Resistance and Response

Biomarkers play a critical role in predicting treatment response and resistance in locally advanced head and neck squamous cell carcinoma (LA-HNSCC). A combination of biomarkers may enhance predictive accuracy and help guide personalized treatment decisions. These include traditional biomarkers such as PD-L1 expression, circulating biomarkers, and image-based methods. 

### 7.1 PD-L1 Expression and Combined Positive Score (CPS)

Programmed death-ligand 1 (PD-L1) is an immune checkpoint protein expressed on tumor and immune cells that suppresses T-cell activity via PD-1 binding. PD-L1 expression is a predictive biomarker of immunotherapy response in head and neck squamous cell carcinoma (HNSCC). The combined positive score (CPS), which quantifies PD-L1 on both tumor and immune cells, offers a broader assessment than the tumor proportion score (TPS). Higher CPS scores generally correlate with an increased likelihood of responding to treatments targeting PD-1/PD-L1 [11]. Phase III trials such as JAVELIN Head and Neck 100 and KEYNOTE-412 did not demonstrate an overall survival benefit with the addition of immune checkpoint inhibitors (ICIs) to chemoradiotherapy in unselected locally advanced HNSCC populations. Exploratory analyses from JAVELIN suggested a possible survival benefit in a subset of patients with high PD-L1 expression, though results were not statistically significant and required validation in larger cohorts. Notably, chemoradiation may upregulate PD-L1 expression, potentially enhancing ICI responsiveness [10,11]. In the GORTEC 2015-01 PembroRad trial, patients were stratified by CPS but later pooled due to limited numbers, resulting in no observed treatment effect differences. The KEYNOTE-048 trial demonstrated improved survival in patients with CPS ≥ 1 or ≥20 treated with pembrolizumab compared to standard chemotherapy [89,90]. Still, inconsistent responses among PD-L1–negative and PD-L1-positive patients highlight the limitations of PD-L1 as a sole predictive biomarker and underscore the need for additional markers.

### 7.2 Emerging Molecular Biomarkers: TMB and MSI

Beyond PD-L1, other biomarkers such as tumor mutational burden (TMB) and microsatellite instability (MSI) are under investigation for their potential to predict response and resistance in locally advanced head and neck squamous cell carcinoma. Tumor mutational burden (TMB) is an emerging biomarker associated with immunotherapy response, leading to FDA approval of pembrolizumab for tumors with TMB ≥ 10 mutations/Mb irrespective of histology. Although high TMB correlates with improved outcomes in various cancers, its predictive value in LA-HNSCC remains unclear. A recent meta-analysis reported better overall response rates and survival in recurrent or metastatic HNSCC patients with high TMB [91], but inconsistent TMB thresholds and small patient cohorts limit the data. Further research is needed to establish clinically relevant cutoffs and validate TMB utility in LA-HNSCC. Microsatellite instability (MSI), resulting from deficient DNA mismatch repair, is a validated biomarker for immune checkpoint inhibitor responsiveness in several malignancies. However, MSI-high status is rare in LA-HNSCC; in one study of 80 advanced cases, only one tumor exhibited MSI-high [92]. Given its low prevalence, routine MSI testing has limited clinical relevance in this context but may be considered in select cases.

### 7.3 Circulating Tumor DNA (ctDNA)

Traditional tissue biomarkers like PD-L1 expression and the combined positive score (CPS) are limited by spatial and temporal tumor heterogeneity, providing only static, site-specific information. In contrast, circulating tumor DNA (ctDNA) offers dynamic, minimally invasive monitoring of tumor burden and molecular changes across diverse tumor subclones. ctDNA can detect minimal residual disease, predict early relapse, and provide real-time prognostic insights. Recent studies have linked post-treatment ctDNA positivity in HPV-negative HNSCC with disease progression and poorer survival [93]. A meta-analysis of over 5000 patients also demonstrated an association with worse overall and progression-free survival [94]. However, ctDNA testing may be limited by low plasma abundance and false positives due to clonal hematopoiesis [95]. Methylation-based ctDNA analysis offers a more stable alternative by targeting consistent cancer-specific DNA methylation changes, making it a promising approach in HNSCC. Nonetheless, cost, availability, and sample requirements remain potential barriers to widespread use.

### 7.4 Imaging Biomarkers

The accuracy of molecular biomarkers may be augmented by advanced imaging modalities, which offer dynamic, multiparametric evaluations of the tumor biology. Positron emission tomography-computed tomography (PET-CT) enables noninvasive stratification and monitoring based on tumor metabolism and hypoxia, with studies demonstrating moderate predictive accuracy [96]. Persistent metabolic activity post-treatment may signal early treatment failure, although false positives and limited specificity remain challenges. Magnetic resonance imaging (MRI), particularly hypoxia-sensitive techniques, can help identify regions of immunotherapy resistance, as hypoxia fosters an immunosuppressive tumor microenvironment. While not yet widely implemented due to cost, accessibility, and lack of standardization, imaging biomarkers offer real-time, noninvasive assessments of treatment response. In a recent multicenter study, a radiomics-clinical nomogram incorporating intra- and peritumoral MRI features outperformed PD-L1 CPS in predicting pathologic complete response to neoadjuvant chemoimmunotherapy in HNSCC [97], supporting the potential of imaging biomarkers to enhance personalized care.

Biomarkers are critical for identifying patients most likely to benefit from immune checkpoint inhibitors (ICIs). However, their role in locally advanced head and neck squamous cell carcinoma (LA-HNSCC) remains underexplored, as most data are derived from studies in the metastatic setting. Given emerging mechanisms of resistance and the inherent limitations of individual biomarkers, a multimodal approach combining molecular, circulating, and imaging-based markers is essential to improve predictive accuracy. While biomarkers offer valuable guidance in treatment selection, their clinical utility in LA-HNSCC is still evolving and requires further validation.

## 8 Future Directions and Emerging Therapies

### 8.1 Novel Immune Checkpoints beyond PD-1/PD-L1 and CTLA-4—Such as TIM-3, TIGIT, LAG-3

Several emerging therapies are set to transform the future treatment landscape for head and neck cancers, particularly with a focus on immune-based approaches. These therapies aim to enhance the host’s immune response against cancer cells. Among the earliest FDA-approved immune checkpoint inhibitors for multiple cancers, including head and neck cancer, are the anti-CTLA-4 antibody ipilimumab and the anti-PD-1 antibody pembrolizumab. However, a significant proportion of tumors show resistance to these checkpoint blockades, leading to increased interest in targeting additional co-inhibitory receptors such as TIM-3, TIGIT, and LAG-3.

T cell immunoglobulin and mucin-domain containing-3 (TIM-3) is a type I transmembrane protein initially identified as a marker of IFN-γ-producing Th1 and Tc1 cells. It plays a crucial role in suppressing Th1 responses and cytokine expression [98]. TIM-3 contributes to tumor progression through various mechanisms, including promoting tumor cell migration and invasion, directly suppressing CD4^+^ T cells via activation of the IL-6/STAT3 pathway to inhibit Th1 polarization, and activating mTOR signaling [98]. Growing evidence supports TIM-3 as a promising immunotherapeutic target, with the potential to broaden the subset of patients who benefit from immune checkpoint therapies.

Immunoreceptor tyrosine-based inhibitory motif domains (TIGIT) represent another novel marker being widely studied in immunotherapy. TIGIT competes with the costimulatory receptor CD226 for binding to the ligand CD155, thereby impairing antitumor immunity by suppressing T cell function. Importantly, TIGIT blockade shows potential to synergize with anti-PD-1 therapies, enhancing overall therapeutic efficacy [95].

Lymphocyte-activation gene 3 (LAG-3) is expressed on activated T and natural killer (NK) cells and serves as an activation marker on both CD4^+^ and CD8^+^ T cells [98]. Emerging studies are exploring the interplay and combined blockade of LAG-3 and PD-1 pathways, as the field advances toward targeting alternative inhibitory receptors to overcome resistance and improve immunotherapy responses.

### 8.2 CAR-T/NK Cell Therapies in Combination with Conventional Treatments

Head and neck cancers are notable for being one of the most complex cancers to treat due to the complex anatomy. Chimeric antigen receptor (CAR) natural killer (NK) cell therapy offers a promising novel approach for advancing treatment in this field. Chimeric antigen receptor (CAR) T/natural killer (NK) cell treatment relies on the modulation of immune cells to identify and destroy cancer cells [99]. This strategy holds potential, especially for patients with high-grade or advanced head and neck cancers. Engineered CAR-NK cells express receptors that bind unique or overexpressed antigens on tumor cell surfaces, enabling precise targeting while retaining the innate cytotoxic capabilities of NK cells to eliminate tumor cells without harming healthy tissue [99]. This ultimately allows effector cells to target a specific antigen more precisely, in addition to maintaining the natural cytotoxicity of NK cells.

Some studies demonstrate that PD-L1-targeted CAR-NK cells effectively eliminate HPV-positive and HPV-negative HNSCC cells, increasing anti-tumor efficacy through IFN-γ production and NK cell activity against tumor cells [99]. Furthermore, research involving localized interleukin-12 (IL-12) expression suggests that combining CAR-NK and CAR-T cells may yield synergistic effects, opening new avenues for combination immunotherapy in HNSCC. However, long-term safety and efficacy data across diverse patient populations remain limited [99]. There is a definite need for more research and clinical evidence of CAR-NK therapy. In spite of this, the advancement in the management of HNSCC is very promising with the above new therapies in mind.

### 8.3 Personalized Immunotherapy Approaches and Adaptive Trial Designs

In the evolving landscape of head and neck cancer (HNC) treatment, there is growing interest in personalized immunotherapy approaches. Research suggests that early-onset HNC—defined as diagnosis before the age of 65—is associated with distinct risk factors and prognostic outcomes, although the underlying biological mechanisms remain largely unclear [100]. This has led to the hypothesis that tailored immunotherapeutic strategies may hold particular promise for patients with early-onset HNSCC, who may exhibit unique tumor biology and microenvironmental characteristics.

Personalized immunotherapy approaches, including defined genetic risk factors such as variation in MICA A5.1, FANCG, CDKN2A, and TP53, offer enhanced effectiveness in the treatment of HNC and may minimize side effects compared with standard therapies [101]. However, these promising findings require validation through larger, well-powered clinical trials to establish their clinical utility.

### 8.4 Targeted Cell Death as an Immunotherapy-Enhancing Strategy: Apoptosis Restoration and IAP Antagonism

An alternative approach to overcoming resistance in LA-HNSCC involves deliberately increasing tumor cell death to promote antigen release, activate innate immune pathways, and facilitate downstream T-cell priming—processes essential for achieving sustained responses to immune checkpoint blockade [102]. Although radiotherapy and platinum-based chemotherapy cause DNA damage, many HNSCC tumors resist cytotoxic effects by favoring survival pathways and inhibiting apoptosis, often through the upregulation of inhibitor of apoptosis proteins (IAPs) such as XIAP and cIAP1/2, which impede caspase activation [103]. Pharmacological inhibition of IAPs using second mitochondria-derived activator of caspases (SMAC) mimetics can restore caspase-mediated apoptosis and, in certain scenarios, activate additional regulated cell death pathways. This targeted intensification of cell death represents a rational strategy to enhance the efficacy of CRT and potentially improve synergy with immunotherapy [103].

Clinical studies involving the oral IAP antagonist xevinapant (Debio 1143) highlight both the potential and the challenges of this strategy. In a randomized phase II trial of unresected LA-SCCHN, adding xevinapant to cisplatin-based CRT yielded promising long-term efficacy, with improved survival observed on extended follow-up. These results support the idea that restoring apoptosis can enhance CRT effectiveness in curative settings [104]. However, the subsequent phase III TrilynX trial showed that xevinapant plus CRT did not improve event-free survival—in fact, EFS was shorter compared to placebo—and the combination was associated with a less favorable safety and tolerability profile [105]. Notably, there were higher rates of serious adverse events and more frequent and earlier chemotherapy dose modifications, suggesting that compromises in treatment delivery may have diminished any biological benefits gained from increased cell death [105]. This issue is particularly important in definitive CRT, where maintaining dose intensity is closely linked to cure rates. Overall, these findings indicate that while targeted cell death is a biologically sound approach, its clinical success in LA-HNSCC likely requires careful optimization of dosing and sequencing, robust supportive care to maintain CRT intensity, biomarker-driven patient selection (such as IAP expression and death pathway competence), and consideration of use in contexts where systemic toxicity is less likely to undermine curative therapy [103].

### 8.5 Surgical Integration as a Determinant of Immunotherapy Success in LA-HNSCC

Recent phase III trials in locally advanced HNSCC indicate that immune checkpoint inhibitors (ICIs) have shown greater therapeutic benefit when integrated into treatment regimens that include surgical resection. In contrast, studies employing definitive chemoradiotherapy (CRT) without surgical intervention have not reliably produced significant improvements in survival outcomes [14,15]. 

The KEYNOTE-689 trial investigated perioperative pembrolizumab across neoadjuvant, concurrent, and adjuvant phases in resectable stage III–IV LA-HNSCC, revealing significant improvements in event-free survival (EFS) and major pathological response, which resulted in FDA approval in 2025 [12,17]. Similarly, the NIVOPOSTOP (GORTEC 2018-01) trial demonstrated a disease-free survival advantage in PD-L1–positive patients receiving adjuvant nivolumab after surgical resection and postoperative CRT [14]. While the IMvoke010 trial did not achieve its primary endpoint of EFS in the overall cohort, it assessed atezolizumab in a high-risk, post-multimodal setting following surgery and/or definitive therapy, supporting the notion that immunotherapy may be more effective in the minimal residual disease setting than in patients with bulky, unresected tumors [12].

Conversely, studies examining the addition of ICIs to definitive CRT without surgery—such as KEYNOTE-412 and JAVELIN Head and Neck 100—did not show statistically significant gains in event-free survival, progression-free survival, or overall survival in unselected patient groups. This disparity highlights several important biological and clinical considerations [10,11].

From a biological standpoint, surgical resection significantly modifies tumor–immune interactions. Removing the primary tumor decreases the overall tumor burden and may eradicate immunosuppressive stromal regions rich in cancer-associated fibroblasts, hypoxic zones, and areas of metabolic competition. The environment following resection is characterized by reduced tumor antigen presence and diminished systemic immunosuppressive signals, potentially making it more favorable for immune checkpoint blockade. Additionally, administering ICIs in the neoadjuvant setting, as done in KEYNOTE-689, may promote T-cell priming when tumor antigen presentation is intact, with surgery subsequently eliminating resistant tumor clones and stromal obstacles [17].

In comparison, definitive CRT for unresected tumors faces challenges such as persistent hypoxia, a dense extracellular matrix, sustained metabolic competition, and ongoing exosomal immune suppression driven by the tumor. Overactivation of DNA damage response pathways during CRT can further restrict immunogenic cell death and reduce the release of tumor antigens, thereby weakening potential synergistic effects with ICIs. In large tumors exhibiting immune exclusion, concurrent use of ICIs may not be adequate to overcome well-established stromal and metabolic resistance mechanisms.

From a clinical perspective, these findings indicate that both timing and disease context play pivotal roles in determining the efficacy of immunotherapy in LA-HNSCC. ICIs appear to be more effective in perioperative or minimal residual disease scenarios, where the tumor burden is lower and immune restoration is more feasible, as opposed to definitive CRT settings, where resistant features of the tumor microenvironment persist. This underscores the importance of biomarker-driven patient selection and the development of sequencing strategies that take into account tumor structure, hypoxia, and immune infiltration characteristics.

Prospective studies should directly compare perioperative and definitive treatment strategies, incorporate translational endpoints that assess immune priming and stromal remodeling, and investigate combination therapies aimed at targeting hypoxia, DNA damage response mechanisms, and CAF-mediated exclusion to enhance outcomes for patients who are not candidates for surgery.

## 9 Conclusion

Locally advanced head and neck squamous cell carcinoma (LA-HNSCC) continues to present a therapeutic challenge despite advancements in surgery, chemotherapy, and radiotherapy. Although the introduction of immune checkpoint inhibitors (ICIs) has shown promise in some patient populations, randomized controlled phase III trials, such as KEYNOTE-412, KEYNOTE-689, JAVELIN Head and Neck, GORTEC REACH, IMvoke010, and NIVOPOSTOP, have yielded underwhelming results in terms of survival benefits when integrated into standard chemoradiotherapy regimens. This is due to treatment resistance arising from complex tumor and host intrinsic interactions such as immunological suppression within the tumor microenvironment (TME), DNA damage repair pathways, hypoxia-driven adaptations, and metabolic reprogramming. Emerging data demonstrate the multifactorial nature of this resistance, with evidence pointing to immunosuppressive cell infiltration, compensatory immune checkpoints, and the failure of effective antigen presentation. The disappointing outcomes of these several large-scale trials reflect not only biological resistance but also the need for improved trial design incorporating prospective biomarker stratification, optimized sequencing of therapies, and standardized chemoradiation backbones.

In response to these challenges in tumor resistance, new therapeutic strategies are being developed. Nanotechnology-driven approaches offer promising tools to improve drug delivery, remodel and alter the TME, and sensitize tumors to both radiotherapy and immunotherapy to enhance efficacy. Likewise, dual inhibition strategies targeting EGFR, HER3, and downstream effectors such as PI3K, AKT, or MET have shown promising results by blocking compensatory signaling at multiple levels. These inhibition strategies, along with tumor biopsies, allow clinicians to target and tailor treatments specific to each patient’s evolving tumor biology. The integration of dynamic biomarkers such as PD-L1 expression, circulating tumor DNA (ctDNA), metabolic signatures, and imaging-based radiomics will also be essential for real-time monitoring and treatment personalization.

A comprehensive analysis of the cited literature and phase III clinical trial data indicates that the most robust evidence supporting clinically meaningful benefits from immune checkpoint inhibition in locally advanced head and neck squamous cell carcinoma (LA-HNSCC) arises predominantly from perioperative or surgery-integrated treatment paradigms and from biologically enriched patient subgroups. In contrast, definitive chemoradiotherapy (CRT)-only strategies applied to unselected populations have not demonstrated similar efficacy. Notably, the KEYNOTE-689 trial provides compelling proof-of-concept that perioperative immunotherapy can enhance clinically relevant outcomes in resectable disease, while results from the NIVOPOSTOP trial suggest that tumors exhibiting PD-L1 positivity may particularly benefit from adjuvant immune checkpoint inhibitor (ICI) therapy following surgical resection and postoperative CRT [14,17]. Conversely, the negative outcomes observed in most definitive CRT-only studies suggest that unresected, bulky tumors may harbor persistent resistance mechanisms—such as hypoxia, metabolic competition, cancer-associated fibroblast (CAF) and extracellular matrix (ECM) mediated immune exclusion, and tumor-intrinsic DNA damage response (DDR) adaptations—that attenuate immunogenic cell death and limit effective immune priming, thereby reducing ICI synergy. Collectively, these findings support a biomarker-driven perioperative therapeutic strategy, augmented by rational combination regimens that target upstream resistance mechanisms (including stromal remodeling, hypoxia/metabolic pathways, and DDR–STING axis activation) and integrated translational endpoints (such as immune infiltration profiles, circulating tumor DNA [ctDNA] kinetics, and hypoxia signatures).

An important limitation in the current literature is the relative lack of prospective stratification by anatomic subsite within head and neck squamous cell carcinoma. Tumors arising from the oral tongue, larynx, oropharynx, and hypopharynx differ substantially in HPV association, mutational landscape, stromal composition, hypoxia burden, and immune infiltration patterns, all of which may influence responsiveness to chemoradiotherapy and immune checkpoint inhibitors. However, most contemporary trials have evaluated heterogeneous cohorts without adequately powered subsite-specific analyses, limiting our ability to draw definitive conclusions regarding localization-dependent benefit. Future studies incorporating systematic anatomic stratification, integrated molecular profiling, and tumor microenvironment characterization will be essential to refine patient selection and advance truly individualized therapeutic strategies in locally advanced HNSCC.

In conclusion, overcoming resistance in LA-HNSCC will require a combination approach that integrates mechanistic understanding, precision diagnostics, novel therapeutic combinations, and personalized, patient-tailored regimens. As our biological and technological toolkit continues to improve, the opportunity to significantly improve outcomes for patients with LA-HNSCC is within reach.

## Data Availability

The raw data supporting the conclusions of this article will be made available by the authors, without undue reservation.

## Abbreviations

LA-HNSCC	Locally Advanced Head and Neck Squamous Cell Carcinoma
HNSCC	Head and Neck Squamous Cell Carcinoma
HNC	Head and Neck Cancer
LA	Locally Advanced
SOC	Standard of Care
ICI(s)	Immune Checkpoint Inhibitor(s)
PD-1	Programmed Cell Death Protein 1
PD-L1	Programmed Death-Ligand 1
TIM-3	T Cell Immunoglobulin and Mucin-domain Containing-3
LAG-3	Lymphocyte Activation Gene-3
TIGIT	T Cell Immunoreceptor with Ig and ITIM Domains
CTLA-4	Cytotoxic T-Lymphocyte–Associated Protein 4
TME	Tumor Microenvironment
TIME	Tumor Immune Microenvironment
TILs	Tumor-Infiltrating Lymphocytes
Tregs	Regulatory T Cells
NK cells	Natural Killer Cells
CAR	Chimeric Antigen Receptor
CAR-T	Chimeric Antigen Receptor T-cell Therapy
CAR-NK	Chimeric Antigen Receptor Natural Killer Cell Therapy
DDR	DNA Damage Response
ATM	Ataxia-Telangiectasia Mutated
ATR	Ataxia-Telangiectasia and Rad3-related
CHK1/2	Checkpoint Kinase 1/2
PARP-1	Poly (ADP-ribose) Polymerase 1
cGAS-STING	Cyclic GMP-AMP Synthase–Stimulator of Interferon Genes
ICD	Immunogenic Cell Death
NER	Nucleotide Excision Repair
ROS	Reactive Oxygen Species
EGFR	Epidermal Growth Factor Receptor
HER2/HER3	Human Epidermal Growth Factor Receptor 2/3
ERBB	Erb-B Receptor Tyrosine Kinase Family
PI3K	Phosphoinositide 3-Kinase
AKT	Protein Kinase B
mTOR	Mammalian Target of Rapamycin
STAT1/STAT3	Signal Transducer and Activator of Transcription 1/3
PTEN	Phosphatase and Tensin Homolog
HIF-1α/HIF-2α	Hypoxia-Inducible Factor 1-alpha/2-alpha
VEGF	Vascular Endothelial Growth Factor
OER	Oxygen Enhancement Ratio
UHDR-RT	Ultra-High Dose Rate Radiotherapy
FLASH-RT	Ultra-High Dose Rate Radiotherapy (FLASH)
CAF(s)	Cancer-Associated Fibroblast(s)
ECM	Extracellular Matrix
iCAF	Inflammatory Cancer-Associated Fibroblast
myCAF	Myofibroblastic Cancer-Associated Fibroblast
FAP	Fibroblast Activation Protein
LDH-A	Lactate Dehydrogenase A
MCT1/4	Monocarboxylate Transporter 1/4
IDO1	Indoleamine 2,3-Dioxygenase 1
A2A/A2B	Adenosine A2A/A2B Receptors
TNF-α	Tumor Necrosis Factor Alpha
IFN-γ	Interferon Gamma
IFN-β	Interferon Beta
IL-6/IL-10/IL-12	Interleukin 6/10/12
TGF-β	Transforming Growth Factor Beta
AHR	Aryl Hydrocarbon Receptor
ctDNA	Circulating Tumor DNA
CPS	Combined Positive Score
TPS	Tumor Proportion Score
TMB	Tumor Mutational Burden
MSI	Microsatellite Instability
dMMR	Deficient Mismatch Repair
CRT	Chemoradiotherapy
RT	Radiotherapy
EFS	Event-Free Survival
PFS	Progression-Free Survival
OS	Overall Survival
DFS	Disease-Free Survival
mPR	Major Pathological Response
ORR	Objective Response Rate
NP(s)	Nanoparticle(s)
LNP	Lipid Nanoparticle
CAPIR	Circulation, Accumulation, Penetration, Internalization, Release
FMT	Fecal Microbiota Transplantation
scRNA-seq	Single-cell RNA Sequencing
HPV	Human Papillomavirus
E6/E7	HPV Oncoproteins
DAMPs	Damage-Associated Molecular Patterns
PET-CT	Positron Emission Tomography–Computed Tomography
MRI	Magnetic Resonance Imaging
TCR	T Cell Receptor
MHC I	Major Histocompatibility Complex Class I
